# Serological and molecular prevalence of *Brucella* spp. among livestock species in Rajasthan, India

**DOI:** 10.3389/fvets.2023.1157211

**Published:** 2023-07-17

**Authors:** Dharm Singh Meena, Lata Sharma, Jyoti Bishnoi, Monika Soni, Nirmal Kumar Jeph, Vikas Galav, Sandeep Kumar Sharma

**Affiliations:** ^1^Centre for Diagnosis, Surveillance and Response of Zoonotic Diseases (CDSRZ), Department of Veterinary Medicine, Post Graduate Institute of Veterinary Education and Research, Jaipur, India; ^2^Department of Veterinary Pathology, Post Graduate Institute of Veterinary Education and Research, Jaipur, India; ^3^Department of Veterinary Microbiology and Biotechnology, Post Graduate Institute of Veterinary Education and Research, Jaipur, India

**Keywords:** *Brucella*, prevalence, seroprevalence, diagnosis, livestock

## Abstract

A seroprevalence and molecular study was carried out in six districts of the state of Rajasthan, India to detect brucellosis in major livestock species. This study involves the testing of 3,245 livestock samples using the Rose Bengal Plate Test (RBPT), Indirect Enzyme-Linked Immunosorbent Assay (i-ELISA), and genus-specific polymerase chain reaction (PCR) markers for molecular diagnosis of the disease. In the tested samples, seroprevalence was 5.06% (CI: 1.96–8.15) using the RBPT test and 6.88% (CI: 1.98–11.78) using the i-ELISA test, while the cumulative seroprevalence (RBPT and i-ELISA) was 3.63% (CI: 0.44–6.83). The prevalence of the disease was 1.27% (CI: 0.56–3.11) when tested using molecular markers. The highest prevalence of brucellosis was detected in Cattle (7.00, 3.22%), followed by camels (5.50, 2.50%), buffalo (2.66, 0.00%), sheep (2.43, 0.41%), and goats (0.58, 0.23%) when serological (cumulative) and molecular diagnosis were considered preferred methods of detection. Cattle (3.22%) and camels (2.50%) also showed a high prevalence of disease when tested using molecular markers. The results of this study reveal that cattle, camel, and sheep brucellosis is prevalent in the study areas.

## 1. Introduction

Brucellosis is an infectious zoonotic disease that is caused by different members of the genus *Brucella* (1–3). The genus consists of 12 known species (4). The six most common species are *B. abortus* (host: cattle), *B. melitensis* (host: sheep, goat), *B. ovis* (host: sheep), *B. suis* (host: pigs), *B. canis* (host: dog), *B. neotomae* (host: wood rat), and *B. microti* (host: common voles) (5–6). A few other pathogenic species of *Brucella* were also isolated from marine animals, e.g.*, B. pinnipedialis*, *B. ceti*, etc. (7). The most devastating prognosis associated with brucellosis is the high rate of abortion and stillbirth in livestock, besides being potentially hazardous to humans (8, 9). Brucellosis not only has global health impacts but also exerts a wide range of socioeconomic disruptions (10). Considering the nearly asymptomatic descriptive epidemiology of the disease, control measures are necessary, and data on its distribution as well as the early and accurate detection of the causative species is the primary requirement to achieve this goal.

This zoonotic disease continues to have considerable economic and public health implications. Many countries around the world have formulated control measures for brucellosis eradication (11). Bovine and caprine brucellosis is primarily endemic in India (IND) (12). The government of India also launched the National Animal Disease Control Program with a projected cost of ₹13,343.00 crores (or ~ $1.64 billion) for foot and mouth disease (FMD) as well as brucellosis during 2019–2024 (13). The brucellosis control program aims to provide 100% vaccination coverage to 3.6 crore female calves with the calfhood vaccine S19 (13, 14). A recent report (2020–21) estimated seropositivity of 8.3% in cattle compared to 5% in 2001 (12, 15–17). The recent increase in cases is substantial; and over the past two decades, the surveillance programs have expanded their coverage to include a larger region, and approximately 30% of the animals have already received vaccinations against brucellosis (18). The prevalence of the disease in sheep and goats is also considerably significant and is estimated to be 11.55 and 5.37%, respectively (19). Since all *Brucella* spp. poses a threat to the health of animals and humans, detection of the bacteria in the early phases of its spread and across all the possible hosts is indeed a necessity.

Rapid laboratory diagnostic procedures for the identification of causative agents play a crucial role in implementing appropriate public health decisions on time. Although bacterial isolation is the “gold standard” in brucellosis diagnosis, it requires certified BSL3 facilities for sample processing and is therefore considered a major bottleneck (20). Serological methods for antigen/antibody detection are employed frequently, and these methods suffer a lower specificity because of cross-reactivity with bacteria closely related to the genus *Brucella* (21, 22). Extensive efforts have been employed to develop a molecular diagnostic assay and to detect the different species (23, 24). The present study was formulated with the objective of using a combinatorial serological approach, viz., the Rose Bengal Plate Test (RBPT) and the indirect-enzyme-linked immunoassay (i-ELISA). The samples were also subjected to a molecular diagnostic test to detect the pathogen in animal blood samples. The present study provides baseline data on brucellosis prevalence in Rajasthan, India (RJ, IND), which will assist in the formulation of a comprehensive program on the descriptive epidemiology of the disease as well as control measures in the state.

## 2. Materials and methods

### 2.1. Ethical approval

(i) Ethical approval for this study was obtained from *National Animal Ethical Committee (Regd. No. 1971/GO/Re/SL/17/CPCSEA; Dated 16 June 2017)*.

### 2.2. Sample collection and processing

**Figure 1:**
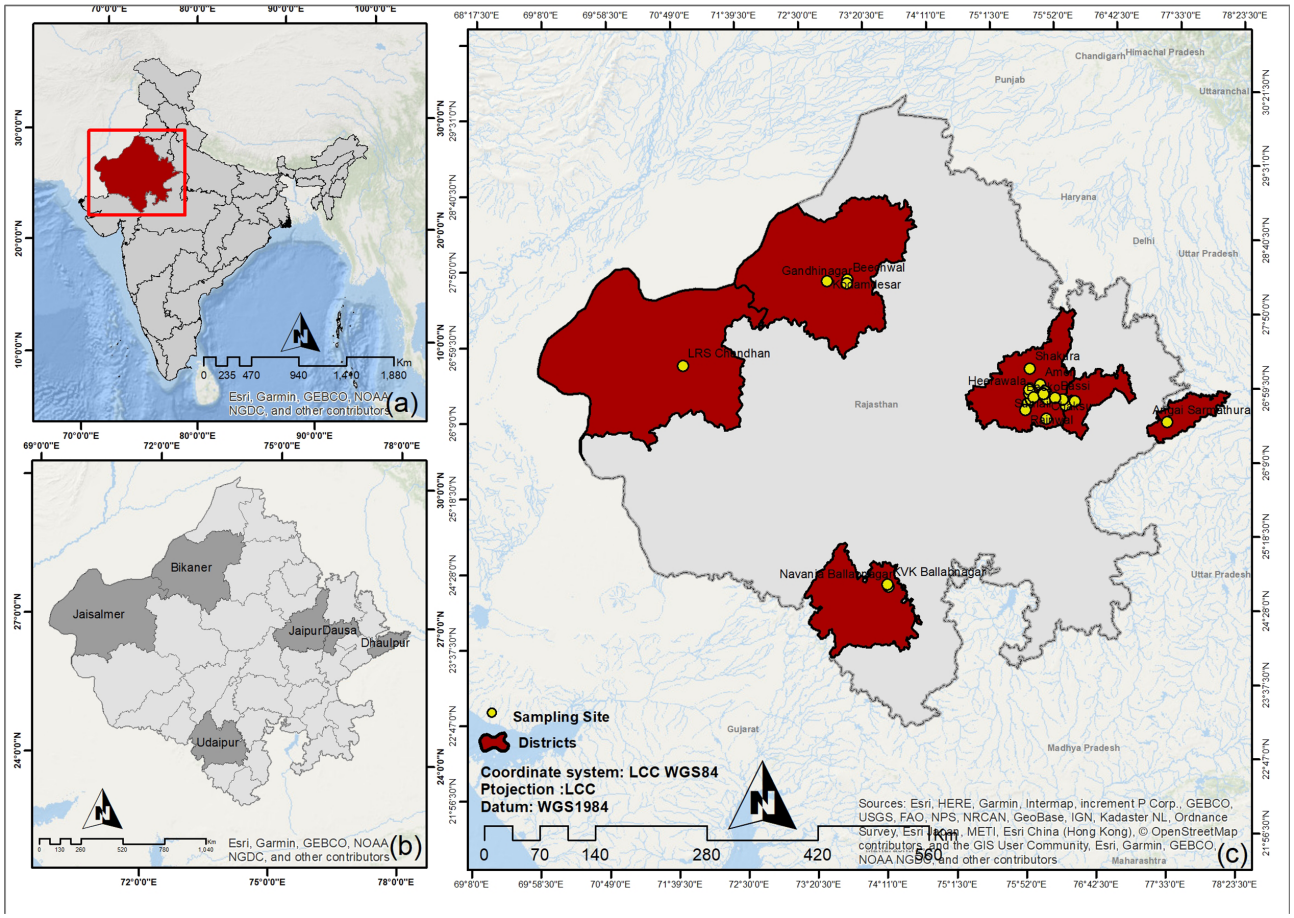
Geographical locations of sample collection sites in Rajasthan. **(A)** National (IND) map. **(B)** State (RJ) map with sampling district highlighted. **(C)** Individual sampling locations.

**Figure 2:**
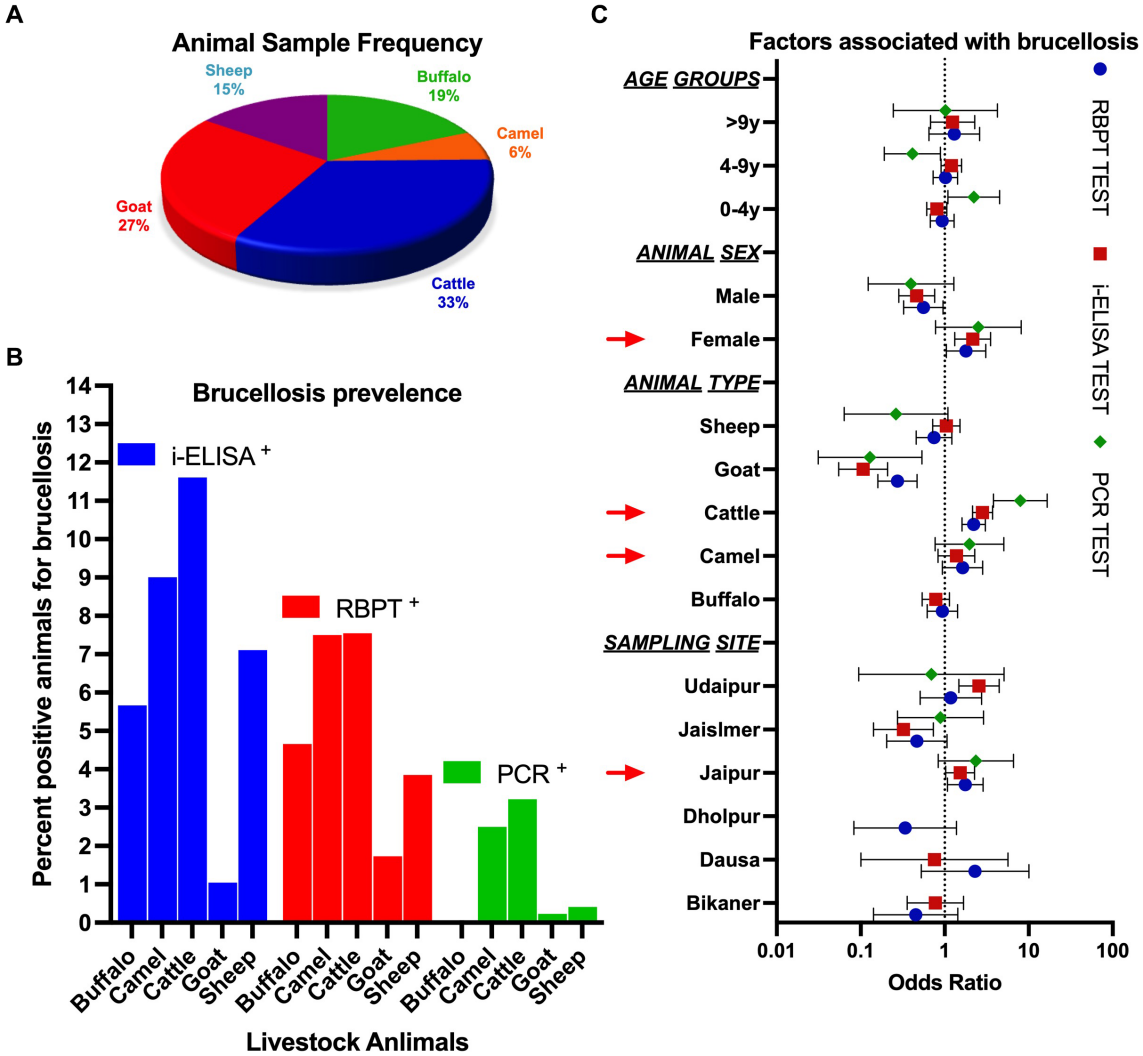
**(A)** Frequency of livestock animals studied under the present study. **(B)** Percentage of animals observed as positive using serological and molecular methods. **(C)** Odds ratio for the likelihood of factors associated with brucellosis.

In the present study, we screened five different livestock animal species of Rajasthan (RJ), India (IND), for the current prevalence and history of brucellosis using serological, immunological, and molecular diagnostic methods. As per the 20th livestock census-2019 estimate by the Department of Animal Husbandry and Dairying, GOI, the state of Rajasthan harbors approximately 20.8 million goats, 13.9 million cattle, 13.7 million buffalo, 7.9 million sheep, and 0.2 million camels (25). As this study was conducted under an active surveillance program, the sample size estimation was not predetermined. Samples were collected randomly from farmed animals. Informed consent was obtained from the animal owners prior to sample collection. In addition to sample collection, a detailed history was recorded for each animal, including any previous diagnoses, treatment history, and exposure to potential sources of infection. At PGIVER, Jaipur, we collected all the samples (non-random sampling) brought to the clinic using the same process as mentioned for the field sampling. For the present study, we collected 3,245 blood samples from six different districts (Bikaner, Dausa, Dholpur, Jaipur, Jaisalmer, and Udaipur) of Rajasthan from July 2020–February 2022 (Figure 1). The sample comprised 1,086 cattle, 865 goats, 601 buffalo, 493 sheep, and 200 camels (Figure 2A). For the surveillance of brucellosis in RJ, animals were randomly selected and properly restrained in the most humane manner by a trained veterinarian. Blood was drawn into a 9 mL vacutainer tube (Greiner bio-one, Austria) by puncturing the jugular vein, followed by proper antiseptic treatment of the animals before they were reverted to their habitat. Samples were labeled with animal identification information (tag number, type, location, sex, age). Epidemiological information regarding the animal and herd level variables was recorded in a questionnaire. Blood samples were transported to the CDSRZ lab for processing. Serum was extracted for the serological test by initially allowing the blood to clot at room temperature in a slanting position followed by centrifugation at 2,000 × g for 10 min. The serum was collected with a pipette and aliquoted into a fresh tube. Serum samples were stored at −20°C until further analysis.

### 2.3. Rose Bengal plate test (RBPT) test

The serum samples were subjected to the Rose Bengal Antigen test (RBPT) (*B. abortus* S99 strain) (IVRI, Izzatnagar, Bareilly, IND) for brucellosis detection according to the recommended method (26). Each test was performed with a proper bovine positive and negative control sera provided by CDSRZ, PGIVER Jaipur. Equal volumes (30 μL) of antigen and test serum were mixed thoroughly on a glass plate using a toothpick, and the mixture was gently agitated or rocked for 3–4 min at room temperature. Any agglutination (observed as spots, flakes, or dotted particles) was considered a positive reaction.

### 2.4. Indirect enzyme-linked immunosorbent assay (i-ELISA) test

An indirect-ELISA (i-ELISA) was performed using a PrioCHECK^®^ BRUCELLA Ab 2.0 ELISA (ThermoFisherSci., United States) in all the serum samples for the detection of IgG antibodies against *Brucella* spp. as per the manufacturer’s protocol. Interpretation of the results was done based on the color development and optical density (OD) values using a Multimode microplate (Tecan, CHE) absorbance reader at 450 nm. The baseline cut-off was set at 40, and any samples below the threshold were reported as negative.

### 2.5. Molecular diagnosis

The clotted blood samples that remained after serum separation were used for DNA isolation in the molecular study. DNA was extracted using the DNASure Blood Mini Kit (Genetix, New Delhi, IND) following the manufacturer’s instructions, with minor modifications. Modifications included the incubation of clotted samples for 1 h at 56°C (overnight at room temperature if sample not lysed for 1 h at 56°C) and high-speed centrifugation at 12000 × g for 3 min to dry column before elution.

#### 2.5.1. Oligonucleotide primers and in-silico analysis

In the present study, we tested the primers from published literature and further designed multiple sets of novel primers through extensive literature and nucleotide sequence searches from the NCBI databases. Primer specificity was analyzed for different species of *Brucella* and checked for any non-specific binding in the host genome (five livestock species sampled for the current study) using Primer blast (27). After optimization with unknown samples and positive control sample, we finally used the following primer sets in the present study IS711 primers (28) and genus-specific primer 1 (GSP-1; designed for the present study as mentioned earlier) (Table 1). The DNA sequence comparisons with the GenBank database were searched and assessed for species or genus assignment using BLAST search (29).

**Table 1:** Primer pairs designed/modified and used for brucellosis diagnosis.

#	Gene/Targets	Primer sequence (5′-3′)		Amplicon length (bp)	Tm (°C)
1.	IS711^a^	Ba-F	GACGAACGGAATTTTTCCAATCCC	492	60
		Bm-F	AAATCGCGTCCTTGCTGGTCTGA		
		R	TGCCGATCACTTAAGGGCCTTCAT		
2.	GSP-1	F	GCAGCTCACGGATAATTTGACC	782	54
		R	ACACCTTGTCCACGCTCAC		

a adopted from Amoupour et al. (28); Ba, *Brucella abortus*; Bm, *B. melitensis*; GSP, genus-specific primer; GSP-1 was designed from a conserved region of the reference sequence of *Brucella abortus* (NC_007624.1).

#### 2.5.2. PCR optimization

The DNA was subjected to PCR detection with suitable adjustments in the concentration of critical reagents, such as primer, MgCl_2_, and template DNA, and annealing temperature of thermocycling to obtain optimal amplification of targeted genes. The PCR was performed using Thermal cyclerProFlex™ (Applied Biosystem, United States) in 10 μL of reaction volume containing 2 μL of 5x Phusion HF buffer (containing 7.5 mM MgCl_2_), 2 U of Phusion Taq DNA polymerase, 200 μM of each dNTP, 5 μM (5pmoles) of each primer (Eurofins Genomics IND Pvt. Ltd., Bengaluru, IND), and 50 ng of genomic DNA. PCR profile consisted of an initial denaturation of 95°C for 5 min, followed by 35 repeated cycles of denaturation at 94°C for 30s, annealing at different temperatures ranging from 54 to 60°C (Table 1) for 30s, extension at 72°C for 1 min, and a final extension at 72°C for 5 min. Then, 5 μL of the PCR product was subjected to electrophoresis on 1.5% agarose gel (Purgene, Genetix, IND) stained by 0.5 μg/mL of ethidium bromide, and the results were evaluated in the presence of GeneRuler 100 bp/1 kb DNA size marker (Thermo-scientific, United Kingdom), visualized under Vilber Fusion solo S Gel documentation system (Eppendorf, DEU). Finally, PCR products were column purified randomly and sequenced in both directions using an ABI 3130 Genetic Analyzer (Applied Biosystem, United Kingdom) with Big Dye Terminator cycle sequencing kit v.3.1 with the help of the same primer (individually) used for amplification of the target gene.

### 2.6. Statistical analysis

The categorical variables, such as age, sex, location of sample collection, type of livestock, etc., were described using counts and percentages. Serological results were presented individually for RBPT and i-ELISA. Cumulative serological results are presented where only the consensus results for both RBPT and i-ELISA were considered true positive. A bivariate analysis was performed to correlate the categorical variables with disease incidence. Pearson’s Chi-square test was used based on the variable size. Logistic regression analysis was performed to delineate the risk factors associated with brucellosis in the livestock. The odds ratio (OR) was calculated along with the confidence intervals (CI) using multiple variables. All the data were analyzed using GraphPad Prism v.9.0 and Microsoft Excel v.2021, and statistical calculations were considered significant with a minimum confidence level of 95% (*p* ≤ 0.05). ArcGIS v. 10.8 was used to prepare the base map of the study area.

## 3. Results

### 3.1. Sample collection

The current study was carried out in RJ, IND between July 2020 and February 2022. A total of 3,245 samples were collected from five livestock species comprising cattle (33.47%), goats (26.66%), buffalo (18.52%), sheep (15.19%), and camels (6.16%) (Figure 2A). Blood samples were collected from six different districts of RJ, which account for ~11.06% of the area covered (Figure 1).

### 3.2. Seroprevalence of brucellosis

The prevalence of brucellosis among livestock comprising cattle (7.55%), camels (7.50%), buffalo (4.66%), sheep (3.85%), and goats (1.73%), and in the studied areas of RJ, it was estimated (mean) to be 5.06% (95% CI: 1.962–8.154) using the RBPT test. Seroprevalence measured using i-ELISA showed the highest prevalence in cattle (11.60%), camels (9.00%), sheep (7.10%), buffalo (5.66%), and goats (1.04%), with a mean prevalence of 6.88% (95% CI: 1.975–11.78) (Figure 2B). Cumulative seroprevalence (RBPT and i-ELISA) was highest in cattle (7.00%), followed by camels (5.50%), buffalo (2.66%), sheep (2.43%), and goats (0.58%). The association of different variables with the prevalence of brucellosis is summarized in Table 2. The RBPT test showed the highest prevalence of the disease in the Dausa district (10.53%), followed by the Udaipur (5.71%) and Jaipur districts (5.32%). The highest seroprevalence of brucellosis detected using i-ELISA was found in the Udaipur district (15.24%), followed by Jaipur (7.29%), Bikaner (5.43%), and Dausa (5.26%), while the samples collected from the Dholpur district were negative for the disease when tested with i-ELISA. Geographical distribution using cumulative serological (RBPT and i-ELISA) screening was observed to be highest in Dausa (5.26%), followed by Udaipur (4.76%), Jaipur (4.18%), Bikaner (1.55%), Jaisalmer (0.81%), and Dholpur (0.00%). Female animals showed a higher prevalence of brucellosis than males; however, the observation could be linked to ~85% of female animals in the sampled population (𝟀^2^ = 9.81^,^ *p* = 0.001). We also observed that the seroprevalence (cumulative) of brucellosis was slightly higher (*p* > 0.05) in animals of 4–9 years of age compared to younger ones (3.71% in the 0.1–4.0 years age group, 3.81% in the 4.1–9.0 years age group, and 2.76% in the >9.0 years group) (Figures 2B,C; for individual prevalence, refer to Table 2).

**Table 2:** Association of different variables with the prevalence of brucellosis.

#	Variables	Brucellosis											
		RBPT+ # (%)	RBPT− # (%)	*χ*^2^ (*p*-value)	i-ELISA+ # (%)	i-ELISA− # (%)	*χ*^2^ (*p*-value)	SERO+ # (%)	SERO− # (%)	*χ*^2^ (*p*-value)	PCR+ # (%)	PCR- # (%)	*χ*^2^ (*p*-value)
1.	Study area												
	Bikaner	3 (2.33%)	126 (97.67%)	9.89^ns^ (0.079)	7 (5.43%)	122 (94.57%)	28.80^****^ (2.54E-05)	2 (1.55%)	127 (98.45%)	142682.36**** (0.00E+00)	0 (0.00%)	129 (100.00%)	4.29^ns^ (0.508)
	Dausa	2 (10.53%)	17 (89.47%)		1 (5.26%)	18 (94.74%)		1 (5.26%)	18 (94.74%)		0 (0.00%)	19 (100.00%)	
	Dholpur	2 (1.75%)	112 (98.25%)		0 (0.00%)	114 (100.00%)		0 (0.00%)	114 (100.00%)		0 (0.00%)	114 (100.00%)	
	Jaipur	140 (5.32%)	2,492 (94.68%)		192 (7.29%)	2,440 (92.71%)		110 (4.18%)	2,255 (95.82%)		40 (1.52%)	2,592 (98.48%)	
	Jaislmer	6 (2.44%)	240 (97.56%)		6 (2.44%)	240 (97.56%)		2 (0.81%)	244 (99.19%)		3 (1.22%)	243 (98.78%)	
	Udaipur	6 (5.71%)	99 (94.29%)		16 (15.24%)	89 (84.76%)		5 (4.76%)	100 (95.24%)		1 (0.95%)	104 (99.05%)	
2.	Livestock species												
	Buffalo	28 (4.66%)	573 (95.34%)	39.11^****^ (6.60E-08)	34 (5.66%)	567 (94.34%)	87.13^****^ (5.36E-18)	16 (2.66%)^$^	585 (97.34%)	47878.59**** (0.00E+00)	0 (0.00%)	601 (100.00%)	50.03^****^ (3.57E-10)
	Camel	15 (7.50%)	185 (92.50%)		18 (9.00%)	182 (91.00%)		11 (5.50%)	189 (94.50%)		5 (2.50%)	195 (97.50%)	
	Cattle	82 (7.55%)	1,004 (92.45%)		126 (11.60%)	960 (88.40%)		76 (7.00%)^$^	1,010 (93.00%)		35 (3.22%)	1,051 (96.78%)	
	Goat	15 (1.73%)	850 (98.27%)		9 (1.04%)	856 (98.96%)		5 (0.58%)	860 (99.42%)		2 (0.23%)	863 (99.77%)	
	Sheep	19 (3.85%)	474 (96.15%)		35 (7.10%)	458 (92.90%)		12 (2.43%)	481 (97.57%)		2 (0.41%)	491 (99.59%)	
3.	Age group												
	0.0–4.0y	94 (4.77%)	1876 (95.23%)	0.61^ns^ (0.738)	124 (6.29%)	1846 (93.71%)	2.44^ns^ (0.295)	73 (3.71%)	1897 (96.29%)	97747.42**** (0.00E+00)	34 (1.73%)	1936 (98.27%)	5.56^ns^ (0.061)
	4.1–9.0y	56 (4.96%)	1,074 (95.04%)		86 (7.61%)	1,044 (92.39%)		43 (3.81%)	1,087 (96.19%)		8 (0.71%)	1,122 (99.29%)	
	>9.0y	9 (6.21%)	136 (93.79%)		12 (8.28%)	133 (91.72%)		4 (2.76%)	141 (97.24%)		2 (1.38%)	143 (98.62%)	
4.	Sex												
	Male	144 (5.25%)	2,600 (94.75%)	4.62^*^ (0.032)	204 (7.43%)	2,540 (92.57%)	9.81^**^ (0.001)	112 (4.08%)	2,632 (95.92%)	155447.97**** (0.00E+00)	41 (1.49%)	2,703 (98.51%)	2.54^ns^ (0.111)
	Female	15 (2.99%)	486 (97.01%)		18 (3.59%)	483 (96.41%)		8 (1.60%)	493 (98.40%)		3 (0.60%)	498 (99.40%)	

SERO – Cumulative serological prevalence represents samples positive for both RBPT and i-ELISA; #-number; %- percentage; ns, non-significant; *- *p* < 0.05, ** < *p* < 0.01, ***- *p* < 0.001, ****- *p* < 0.0001; ^$^ – Due to ongoing vaccination program on brucellosis, the results of seroprevalence could be affected by vaccination status of < 4 y cattle (1.75%) and buffalo (1.33%) considering the vaccination was 100% in those animals.

### 3.3. Molecular detection of brucellosis

We estimated an average of 1.27% brucellosis prevalence among livestock using molecular methods. Intriguingly, the highest prevalence was detected in cattle (3.22%), followed by camels (2.50%), while buffalo were found to be negative for the disease when detected using molecular methods (Figure 2B). Although we could amplify the designed primers in the positive control DNA provided by Dr. D.K. Singh, Principal Scientist, IVRI, Bareilly, IND, species-specific primers (data not provided) could not be amplified in the samples (except IS711) diagnosed based on prognostic history as well as serological tests and, therefore, only genus-specific primers (GSP) were used for diagnostic inference (Table 1). We amplified GSP-1 and IS711 for the detection of brucellosis and sequenced a few random samples to detect the specificity of the primers. Sequenced samples were assembled in CLC workbench v.10 and blasted against the available database in NCBI. Sequence analysis showed the highest similarity with *Brucella* or other congeneric bacterial species.

## 4. Discussion

Brucellosis remains a major infectious disease of livestock and a re-emerging zoonotic disease in several developing countries including India (30). To our knowledge, this is the first epidemiological study comprising five major livestock species (n = 3,245 samples) and covering ~11.06% of the area of RJ. Therefore, the study conducted using the serological and molecular diagnostic assays will provide baseline data on brucellosis prevalence in RJ, IND, and will assist in the formulation of comprehensive disease control measures and eradication programs in the area (Odds ratio in Figure 2C). Priyanka et al. (31) previously reported the presence of *B. abortus* in buffalo in Western Rajasthan. The study was non-randomized and conducted on samples with a plausible history of the disease and was, therefore, excluded from any comparison with the current study. Soni et al. (32) carried out the brucellosis surveillance in buffalo samples in Kota, RJ, and observed a very high prevalence of 35.09% (153/436 samples) using the RBPT test. There are several reports from other countries where brucellosis prevalence ranged from low to moderate. Selim et al. (33) found that in Egypt, the seroprevalence of brucellosis was 16.7% in cattle and 16.25% in sheep. Okafor et al. (34) reported a higher seroprevalence of 38 and 10% in cattle using the RBT and cELISA tests, respectively. Khan et al. (35) estimated the seroprevalence of brucellosis in camels using four serological methods, with RBPT, i-ELISA, c-ELISA, and CFT identifying 15.5, 22.8, 20.2, and 31.0% positive samples, respectively. These estimates are much higher than reported in the present study. Mohamud et al. (36) used competitive-ELISA and found that the camel population in the Puntland State of Somalia had a substantial 7% prevalence of brucellosis. In contrast, Elderbrook et al. (37) reported a low animal seroprevalence of 0.53% in domestic sheep in Wyoming, United States. Shi et al. (6) conducted a meta-analysis and estimated that the overall seroprevalence of buffalo brucellosis worldwide was 9.7%. Comparatively, the seroprevalence of brucellosis in buffalo reported in the present study is much lower than the average worldwide prevalence.

Serological methods are preferable and enable the testing of a high number of samples in a shorter period. Lukambagire et al. (38) noted a high diagnostic accuracy of different serological tests, such as RBPT (~95.9%) and competitive ELISA (~89.4%) for the brucellosis diagnosis. Pabuccuoglu et al. (39) evaluated the specificity of serological tests of brucellosis using pre-diagnosed patients (humans with acute, subacute, or chronic brucellosis) and found that the RBPT test was highly specific (95.7%) for the diagnosis. Indirect-ELISA (i-ELISA) also has high specificity for brucellosis diagnosis (i-ELISA) from milk and serum samples and was found to be 99.1% specific for disease diagnosis (40). Mainar-Jaime et al. (41) also proposed that i-ELISA used alone may be more adequate than the classical RBPT/CFT or any other combinations for brucellosis diagnosis. However, these recommendations were proposed for an area with low-prevalence or brucellosis-free areas (41). In the current study, we did not evaluate the specificity of the tests, but as noted previously, the choice of serological methods used for the current study was rational. We detected the highest seroprevalence of brucellosis in cattle (7.00%) followed by camels (5.50%), buffalo (2.66%), sheep (2.43%), and goats (0.58%) when RBPT and i-ELISA (in combination) were considered preferable methods of detection. Deka et al. (42) detected much higher seropositivity in urban areas (18.7%) compared to rural areas (12.4%) from the state of Assam, IND. Shakuntala et al. (43) reported an overall prevalence of 6.4% by RBPT and 10.7% using ELISA in Meghalaya, IND. Holt et al. (44) reported 15.10% disease prevalence in animals (cattle and buffalo), while Mangtani et al. (45) reported 2.24% positivity in humans in the state of Punjab, IND. Seroprevalence (using RBPT) in sheep was reported to be 23.70% in unorganized sectors and 4.06% in organized sectors of the state of Gujarat, IND (46). Natesan also observed a higher prevalence of brucellosis in sheep (8.29%) in the state of Karnataka. Sonekar et al. (47) analyzed the samples collected from migratory sheep flocks with a history of abortions in the state of Maharashtra, IND. The samples from these flocks showed an alarming 43.31 and 66.24% positive samples through RBPT and ELISA, respectively (47). Comparatively, the prevalence of brucellosis estimated in the current study is much lower (3.85% using the RBPT test and 7.10% using i-ELISA) in the sheep population of RJ, IND. Sheep that are preferentially kept in very dense flocks and are therefore susceptible animals become infected more often through direct contact with infected animals. Frequent surveillance, tagging of animals, and keeping infected animals away from the rest of the flocks could be a better strategy to avoid disease spread.

Currently, there is a lack of common agreement on the usage of molecular markers as different researchers used separate targets, and it has been emphasized that each evaluated method might be suitable for a specific sample type (48). We attempted to evaluate the molecular markers reported by different authors; however, a contradiction or failure to reproduce them limits the applicability of these markers. The primers designed in the current study, along with IS711 (28), were able to diagnose brucellosis in 1.27% (CI: 0.56–3.11) of animal samples (1.05% samples were positive for at least one serological and molecular test). The designed primers successfully amplified in the positive control (*B. abortus* DNA provided by IVRI, Bareilly), and the amplicons of unidentified samples sequenced using Sanger sequencing confirmed the applicability of these markers. Different genes may vary in sequence and amplicon sizes due to InDel, variations, and lengths of amplicon (in the case of IS711), even in different strains of the same species. Therefore, the validation of the specificity and sensitivity of molecular targets is required before proposing them for broader surveillance programs. Molecular methods including PCR (Multiplex, end-point, and real-time) (23, 49), loop-mediated isothermal amplification (24), and lateral flow assay (50), etc. have been employed to diagnose brucellosis in livestock and humans. In the present study, the samples detected as positive using molecular assay were randomly sequenced for IS711 and GSP-1, which revealed the closest similarity with *Brucella* spp. Therefore, the primers used in the current study can detect *Brucella* at the genus level. Based on molecular detection, cattle (3.22%) and camels (2.50%) showed the highest prevalence of the disease. In RJ, camels are one of the most important livestock animals and a major source of income for the nomadic population. They are also used for riding and safaris and, therefore, transmission of zoonotic brucellosis to humans is also possible. Rajasthan currently comprises ~84% of the camels in India (51) and, therefore, a dedicated program is required to screen and vaccinate this animal for the disease. Bansal et al. (52) indicated an alarming seroprevalence (~16.7%) of brucellosis (likely through zoonosis) in human samples in RJ. The present study, along with Bansal et al. (52), does not emphasize the transmission of disease from camels or any other studied animal, but the eradication of the disease will surely require targeting all the possible hosts simultaneously. The current study confirms a marginal prevalence of brucellosis in the cattle, camel, and sheep livestock of Rajasthan, IND, which is lower than that of earlier reports from the state. We also recommend dedicated surveillance for camelids in Rajasthan.

## 5. Future recommendations

The current study highlights a pitfall in sample collection. Although blood sample collection is a gold standard for ELISA-based detection methods and can also be used for molecular detection, farmers/farm owners are not always receptive to this methodology, therefore causing a major bottleneck for the collection of the samples. On the other hand, milk or fecal samples may not be suitable for serological and/or molecular detection in chronically affected animals. Standardization of a common protocol and methodology in a pilot study and a sampling technique keeping the receptiveness of farm owners in consideration is highly warranted. We also accentuate the requirement of the development of a molecular detection method and a common protocol comprising a combinational use of serological and molecular methods (for genus as well as species-specific targets) for the detection of *Brucella* species in livestock animals.

## Data availability statement

The original contributions presented in the study are included in the article/supplementary files. Further inquiries can be directed to the corresponding author.

## Ethics statement

The animal study was reviewed and approved by Ethical approval for this study was obtained from *Institutional Animal Ethical Committee (Regd. No. 1971/GO/Re/SL/17/CPCSEA; Dated 16 June 2017)*. Written informed consent was obtained from the owners for the participation of their animals in this study.

## Author contributions

DM, NJ, VG, and SS: study design and manuscript review. LS and DM: manuscript writing and data analysis. LS, JB, and MS: sample collection, processing, and analysis. All authors contributed to the article and approved the submitted version.

## Funding

The current study was funded by Rastriya Krishi Vikas Yojana (RKVY).

## Conflict of interest

The authors declare that the research was conducted in the absence of any commercial or financial relationships that could be construed as a potential conflict of interest.

## Publisher’s note

All claims expressed in this article are solely those of the authors and do not necessarily represent those of their affiliated organizations, or those of the publisher, the editors and the reviewers. Any product that may be evaluated in this article, or claim that may be made by its manufacturer, is not guaranteed or endorsed by the publisher.
