# Corrigendum: Serological and molecular prevalence of *Brucella* spp. among livestock species in Rajasthan, India

**DOI:** 10.3389/fvets.2023.1275306

**Published:** 2023-08-24

**Authors:** Dharm Singh Meena, Lata Sharma, Jyoti Bishnoi, Monika Soni, Nirmal Kumar Jeph, Vikas Galav, Sandeep Kumar Sharma

**Affiliations:** ^1^Centre for Diagnosis, Surveillance and Response of Zoonotic Diseases (CDSRZ), Department of Veterinary Medicine, Post Graduate Institute of Veterinary Education and Research, Jaipur, India; ^2^Department of Veterinary Pathology, Post Graduate Institute of Veterinary Education and Research, Jaipur, India; ^3^Department of Veterinary Microbiology and Biotechnology, Post Graduate Institute of Veterinary Education and Research, Jaipur, India

**Keywords:** *Brucella*, prevalence, seroprevalence, diagnosis, livestock

In the published article, an author's name was incorrectly written as Dhram Singh Meena. The correct spelling is Dharm Singh Meena.

The authors apologize for this error and state that this does not change the scientific conclusions of the article in any way. The original article has been updated.

